# Supplementary material for: CRISPR/Cas9-mediated glycolate oxidase disruption is an efficacious and safe treatment for primary hyperoxaluria type I
Source: Nat Commun. 2018 Dec 21;9:5454. doi: 10.1038/s41467-018-07827-1 (PMC6303323; doi:10.1038/s41467-018-07827-1)
Supplement: Supplementary file 1 — Supplementary Information [file 41467_2018_7827_MOESM1_ESM.pdf]

Supplementary information

CRISPR/Cas9-mediated glycolate oxidase disruption is an efficacious and safe treatment for primary hyperoxaluria type I.

Zabaleta et al.

Supplementary Figure 1.

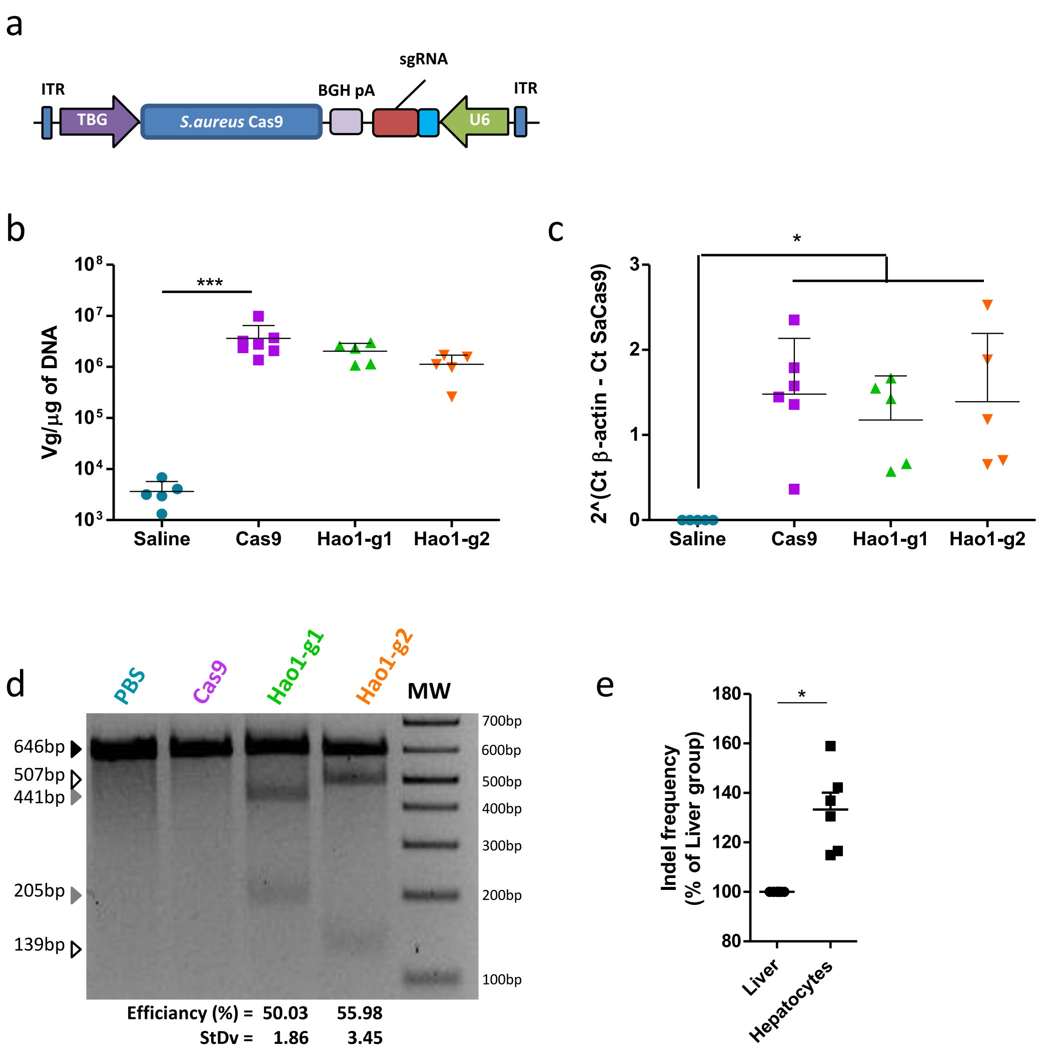

Supplementary Figure 1. Efficient *in vivo* GO inhibition using CRISPR/Cas9. (a) Schematic representation of the vector used in this study. ITR: Inverted Terminal Repeat; TBG: Thyroxine binding globulin promoter; BGH pA: Bovine growth hormone poly A signal; sgRNA: single-guide RNA; U6: U6 promoter. (b) Quantification of viral genome copies by qPCR in the liver of 12-14-week-old PH1 animals treated with saline (n=5), Cas9 (n=6), *Hao1*-g1 (n=5) and *Hao1*-g2 (n=5), one month after the administration of the treatment. (c) SaCas9 mRNA expression by RT-qPCR in the livers as in b. Data in b and c are presented as mean ± SEM and Kruskal Wallis statistical test was used to evaluate differences between groups. (d) Analysis of indels in the liver of animals treated with saline, Cas9, *Hao1*-g1 and *Hao1*-g2 (one representative animal per condition) by Surveyor assay. Black arrow indicates the size of the unmodified PCR band, and grey and white arrows the band size of the cleavage products for *Hao1*-g1 and *Hao1*-g2 respectively. (e) Increase of the frequency of CRISPR/Cas9 introduced indels in the *Hao1* gene analyzed by NGS in purified hepatocytes versus whole liver extracts from 12-14-week-old PH1 animals, treated with *Hao1*-g1 (n=3) and *Hao1*-g2 (n=3). Mann Whitney test was used to compare groups. *p<0.05; ***p<0.001.

Supplementary Figure 2.

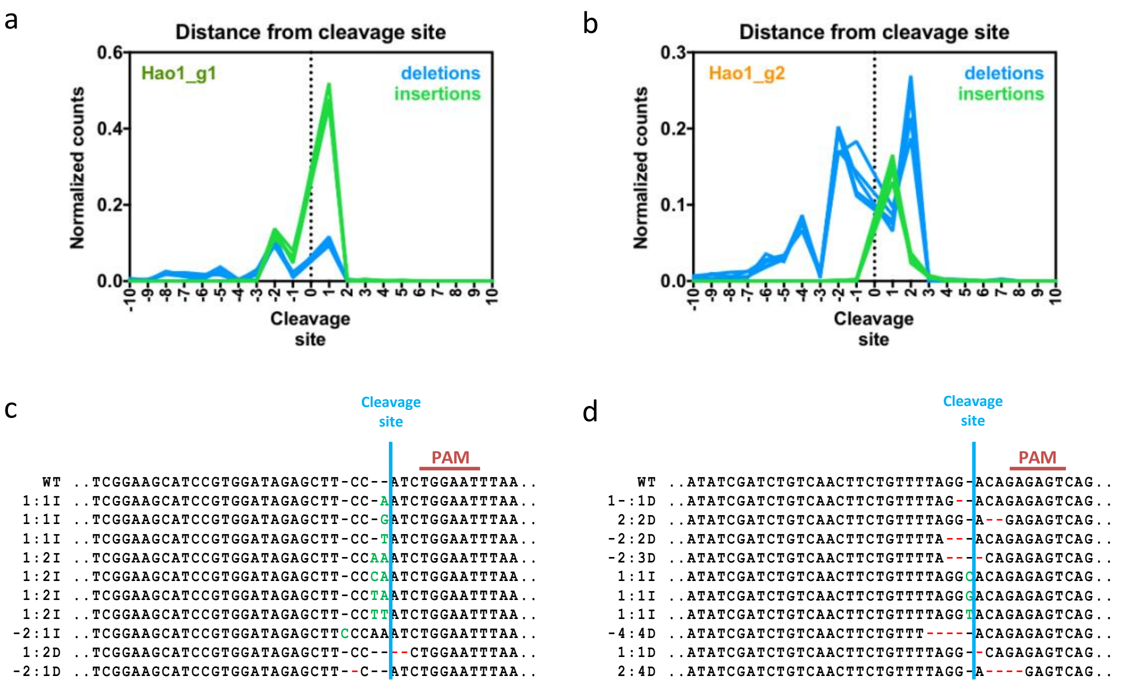

Supplementary Figure 2. Characterization of CRISPR/Cas9-mediated *Hao1* gene editing. Deep sequencing was performed on the DNA from livers of 12-14-week-old PH1 animals treated with *Hao1*-g1 (n=5) and *Hao1*-g2 (n=5), 1 month after treatment. (a and b) Distance from cleavage site of the insertions (green) and deletions (blue) generated by *Hao1*-g1 and *Hao1*-g2 vectors. Each line represents an individual mouse. (c and d) Top 10 indels generated in the liver of a representative mouse by *Hao1*-g1 and *Hao1*-g2 vectors. Green letters indicate inserted nucleotides and red dashes deleted nucleotides. Shaded green and shaded orange indicate *Hao1*-g1 and *Hao1*-g2 binding sites respectively.

Supplementary Figure 3.

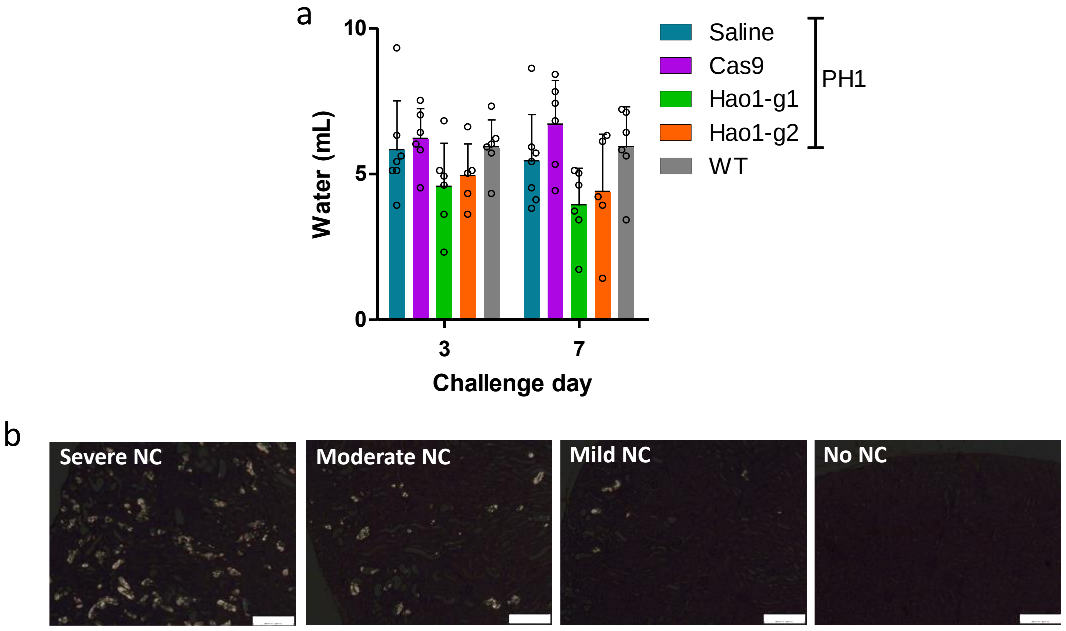

Supplementary Figure 3. Therapeutic efficacy of CRISPR/Cas9 mediated STR in PH1 animals. (a) Measurement of water intake (milliliters) in 8-10-week-old PH1 animals treated with saline (n=7), Cas9 (n=6), *Hao1*-g1 (n=6) and *Hao1*-g2 (n=5) at days 3 and 7 of EG challenge performed 4 months after treatment. Data are presented as mean ± SEM. Friedman test was no significant. (b) Representative digitalized pictures of kidneys presenting various degrees of nephrocalcinosis (NC) used to quantify percentage of NC area (area occupied by calcium oxalate crystals). No NC (0%); Mild NC (0-0.99%); Moderate NC (1-5%); Severe NC (>5%). Scale bar: 200 μm.

Supplementary Figure 4.

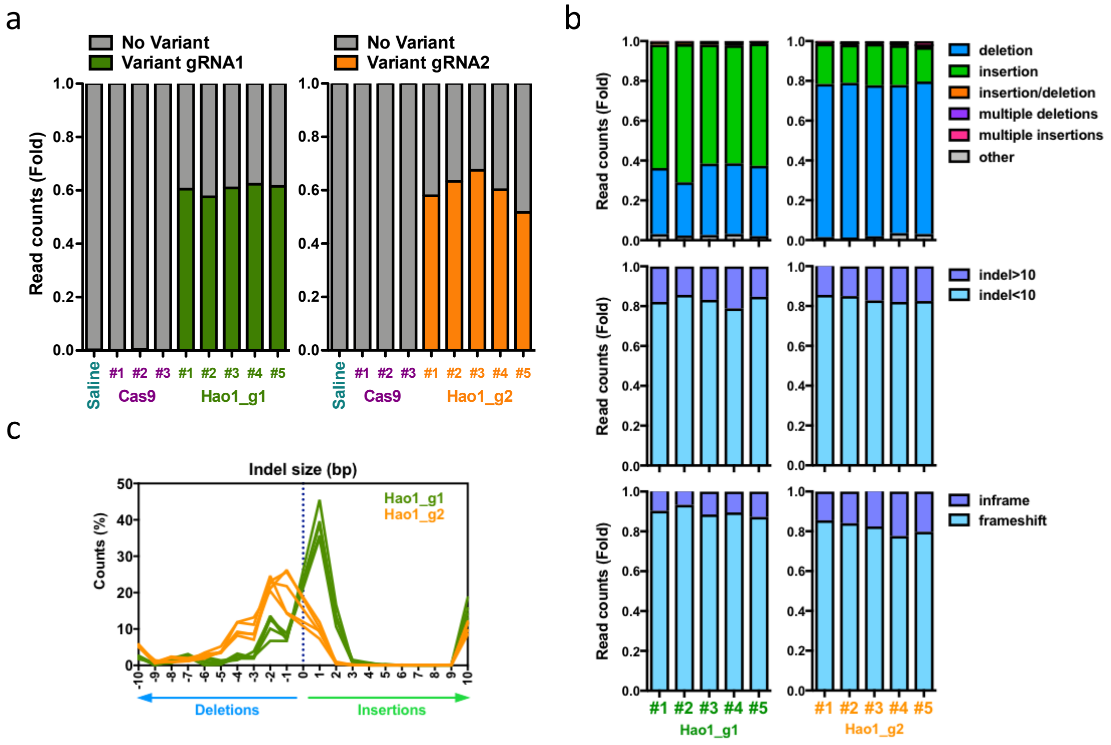

Supplementary Figure 4. Deep sequencing of *Hao1* gene 6 months after treatment. (a) Frequency of CRISPR/Cas9 introduced variants in *Hao1* gene in 8-12-week-old PH1 animals treated with the therapeutic vectors *Hao1*-g1 (n=5) and *Hao1*-g2 (n=5), as well as control vectors (Cas9 n=3 and saline n=1). (b) Characterization of the variants according to their type, size and frameshift potential in animals treated with *Hao1*-g1 and *Hao1*-g2 vectors. (a and b) Each bar represents an individual mouse. (c) Frequency distribution of indel size in base pairs (bp) for each sgRNA in animals treated with *Hao1*-g1 (green lines) and *Hao1*-g2 (orange lines) vectors. Each line represents an individual mouse.

Supplementary Figure 5.

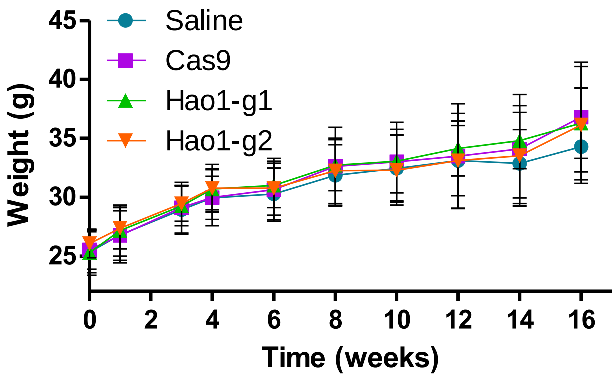

Supplementary Figure 5. Safety of the expression of CRISPR/Cas9 system. (a) Evolution of animal weight during 4 months after vector administration before EG challenge. Saline (n=7), Cas9 (n=6), *Hao1*-g1 (n=6) and *Hao1*-g2 (n=5). No significance was found in Friedman test.

Supplementary Figure 6.

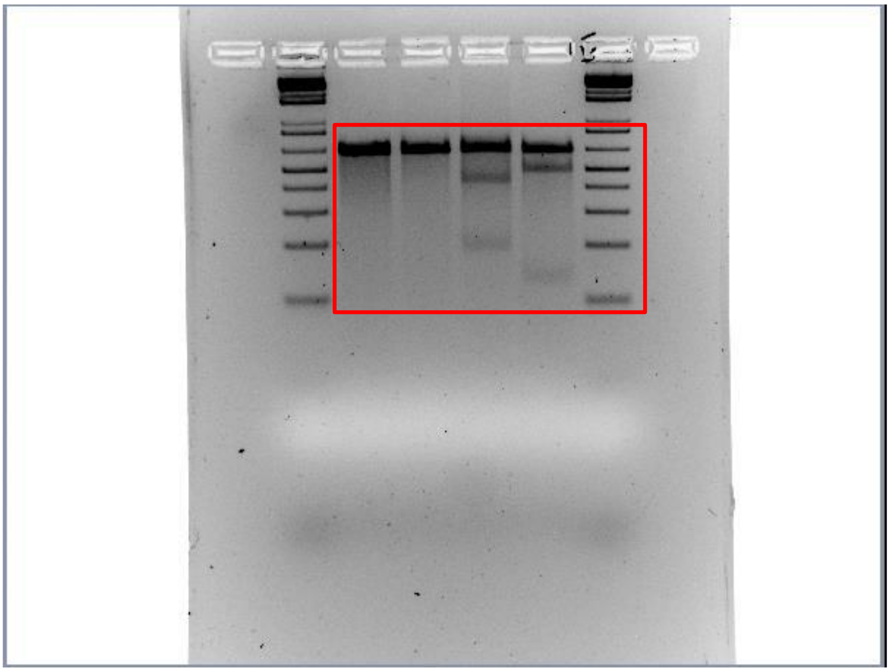

Supplementary Figure 6. Raw electrophoresis gel of Supplementary Figure 1d. Red square indicates image showed in Supplementary Figure 1d.

Supplementary Figure 7.

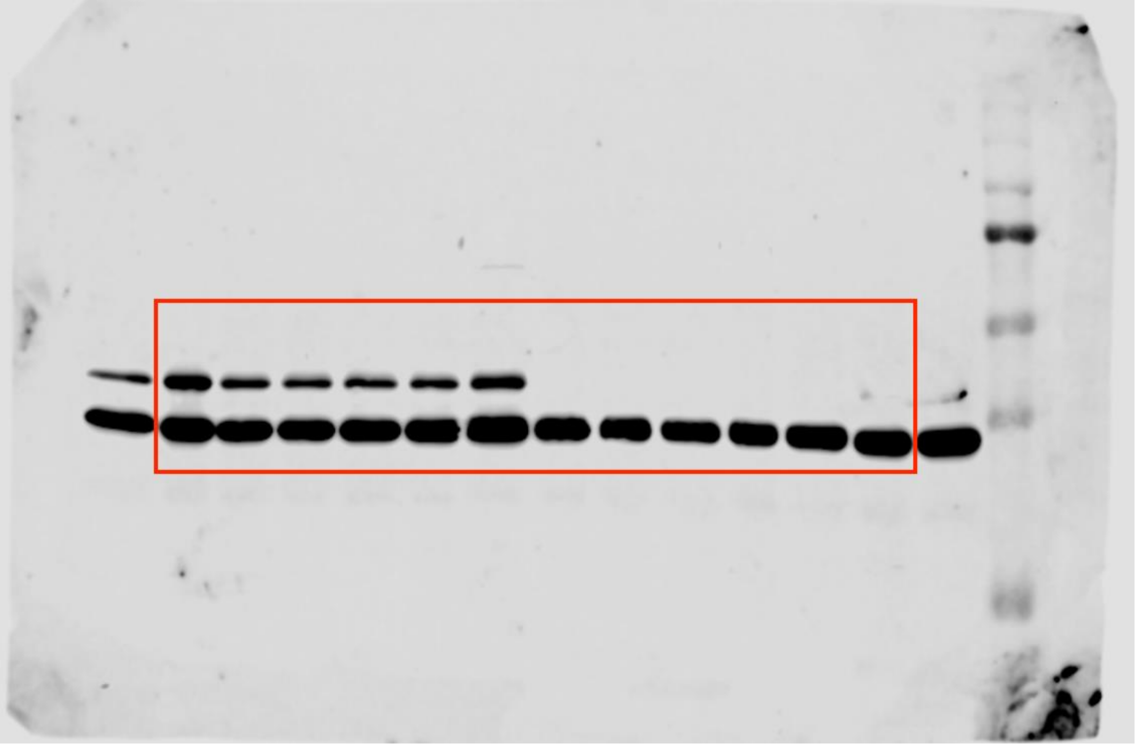

Supplementary Figure 7. Raw western blot of Figure 1d obtained by using Odyssey Fc (LI-COR) imaging system. Red square indicate image showed in Figure 1d.

Supplementary Table 1: Design of gRNAs against mHao1 exonic region.

Name	Hao1-g1	Hao1-g2
Taget gene	mouse Hao1 gene	mouse Hao1 gene
Region	Exon 2	Exon 2
DSB position	6075	6139
Direction	Antisense	Sense
Sequence	TCCGTGGATAGAGCTTCCATC	GTCAACTTCTGTTTTAGGACA
On-target score	51.8[1]	45.1[1]
Off-target score	87.8[2]	65.5[2]

[1]Algorithm published by Doench et al. Nat. Biotechnol. 32, 1262–1267 (2014).
[2]Algorithm published by Hsu et al. Nat. Biotechnol. 31, 827–832 (2013).

Supplementary Table 2: Off-Targets of *Hao1*-sgRNAs.

gRNA	OFT	Sequence	PAM	Score	M	Chr	St	Start	End	Cut
Hao1-g1	OFT1	ATAGGGGAAAGAGCTTCCATC	TGGAG	1.49	5	chr8	-	70465838	70465858	70465841
	OFT2	AGTGTGGGCAGAGCTTCCATC	AAGAG	1.04	5	chr8	-	114836217	114836237	114836220
	OFT3	CACGGGGACAGAGCTTCCATT	TGGAA	0.91	5	chr14	-	99875996	99876016	99875999
	OFT4	TCCACGGATACAGCTTCCATT	ATGAG	0.79	4	chr3	-	53630936	53630956	53630939
	OFT5	TCCTGGGACAGAACTTCCATC	CAGAA	0.77	4	chr18	+	38602949	38602969	38602967
	OFT6	TTTGTAGATAGAGTTTCCATC	TGGAA	0.67	4	chr1	+	187771905	187771925	187771923
	OFT7	AAAGAGGATAGAGTTTCCATC	CTGAG	0.66	5	chr5	+	98852720	98852740	98852738
Hao1-g2	OFT1	TCACACTTCTGTTTTAGGACA	AAGAA	2.32	4	chr2	+	51639105	51639125	51639123
	OFT2	TTGACTTTATGTTTTAGGACA	AAGAG	1.44	5	chr10	+	112299338	112299358	112299356
	OFT3	CTCACCTTCTCTTTTAGAACA	TTGAA	1.24	4	chr3	+	138832512	138832532	138832530
	OFT4	CACAGTTCCTGTTTTAGGACA	TGGAG	0.99	5	chr14	-	68699401	68699421	68699404
	OFT5	GTCAAATATTATTTTAGGACA	CAGAA	0.89	4	chr10	-	50197631	50197651	50197634
	OFT6	ATAAAGTTCATTTTTAGGACA	TAGAA	0.88	5	chr1	-	171651706	171651726	171651709
	OFT7	ATGAAAATGTGTTTTAGGACA	GGGAG	0.87	5	chr6	-	66364482	66364502	66364485

OFT: off-target. M: Mismatches. Chr: Chromosome. St: Strand.

[1]Algorithm published by Doench et al. Nat. Biotechnol. 32, 1262–1267 (2014).

Supplementary Table 3: Average percentage of variants in on-target region at different tissues.

Target Site	Time after treatment	Group	Brain	Heart	Kidney	Lung	Spleen
Hao1-g1	6 months	Saline (n=3)	0.073	0.083	0.060	0.080	0.083
		Cas9 (n=4)	0.070	0.077	0.070	0.095	0.067
		Hao1-g1 (n=5)	0.082	0.088	0.068	0.090	0.082
Hao1-g2	6 months	Saline (n=3)	0.056	0.073	0.056	0.073	0.050
		Cas9 (n=4)	0.062	0.070	0.047	0.077	0.052
		Hao1-g2 (n=5)	0.054	0.074	0.058	0.090	0.062

Supplementary Table 4. List of primers used in this study.

Name	Sequence	Use
mHao1_g1_ONT_F	ACACGACGCTCTTCCGATCTagaccaatgtttgtcagagg	NGS
mHao1_g1_ONT_R	CTGGAGTTCAGACGTGTGCTCTTCCGATCTAGTAGCCCCAACACATATTG	NGS
mHao1_g1_OF1_F	ACACGACGCTCTTCCGATCTGAACTTGCTTTGTGGATCAG	NGS
mHao1_g1_OF1_R	CTGGAGTTCAGACGTGTGCTCTTCCGATCTCATGGCATATGTATGTGCAC	NGS
mHao1_g1_OF2_F	ACACGACGCTCTTCCGATCTGACATGGGGAATTTTGATCG	NGS
mHao1_g1_OF2_R	CTGGAGTTCAGACGTGTGCTCTTCCGATCTGTGGGGATTTTTCAAACTCG	NGS
mHao1_g1_OF3_F	ACACGACGCTCTTCCGATCTAAGAACTGTGTAACCTGACC	NGS
mHao1_g1_OF3_R	CTGGAGTTCAGACGTGTGCTCTTCCGATCTCCCCATCATTGTAGTCCATT	NGS
mHao1_g1_OF4_F	ACACGACGCTCTTCCGATCTTTCAGACAGCTCCTTTGTAC	NGS
mHao1_g1_OF4_R	CTGGAGTTCAGACGTGTGCTCTTCCGATCTggccattgtattgagggtat	NGS
mHao1_g1_OF5_F	ACACGACGCTCTTCCGATCTAACCCTCAGACTTAGTCTCA	NGS
mHao1_g1_OF5_R	CTGGAGTTCAGACGTGTGCTCTTCCGATCTTTACTTGTCCTTCTGGTCAC	NGS
mHao1_g1_OF6_F	ACACGACGCTCTTCCGATCTcaggccactttagtttgttg	NGS
mHao1_g1_OF6_R	CTGGAGTTCAGACGTGTGCTCTTCCGATCTttatcttcctgctgacacag	NGS
mHao1_g1_OF7_F	ACACGACGCTCTTCCGATCTGTGCAGTGGAAATATGAACC	NGS
mHao1_g1_OF7_R	CTGGAGTTCAGACGTGTGCTCTTCCGATCTCCTCTCTGCTTTTTCTCAGT	NGS
mHao1_g2_ONT_F	ACACGACGCTCTTCCGATCTcagATGGAAGCTCTATCCAC	NGS
mHao1_g2_ONT_R	CTGGAGTTCAGACGTGTGCTCTTCCGATCTtaaaagcatcctaggaaggg	NGS
mHao1_g2_OF1_F	ACACGACGCTCTTCCGATCTCTCATCTTCAGCTCCTTACC	NGS
mHao1_g2_OF1_R	CTGGAGTTCAGACGTGTGCTCTTCCGATCTTTGCTCTAACTTAGCTGTGG	NGS
mHao1_g2_OF2_F	ACACGACGCTCTTCCGATCTCAGCAACCTTGAACAAGTAC	NGS
mHao1_g2_OF2_R	CTGGAGTTCAGACGTGTGCTCTTCCGATCTGCCACCTGAGTGCTATTTTA	NGS
mHao1_g2_OF3_F	ACACGACGCTCTTCCGATCTTCTTCTCTGGAGTCTGAGTC	NGS
mHao1_g2_OF3_R	CTGGAGTTCAGACGTGTGCTCTTCCGATCTGAAAAGGTCAGGACAGTCAG	NGS
mHao1_g2_OF4_F	ACACGACGCTCTTCCGATCTGGTAGACCATCTGAGTCCTA	NGS
mHao1_g2_OF4_R	CTGGAGTTCAGACGTGTGCTCTTCCGATCTGAAGGAGGAAGCAATTGTTG	NGS
mHao1_g2_OF5_F	ACACGACGCTCTTCCGATCTACCAGAGAGCTTCCTGATAT	NGS
mHao1_g2_OF5_R	CTGGAGTTCAGACGTGTGCTCTTCCGATCTTGCTTAAAAACCATGAAGGC	NGS
mHao1_g2_OF6_F	ACACGACGCTCTTCCGATCTGGGTTCTGTCCAAGATTTCT	NGS
mHao1_g2_OF6_R	CTGGAGTTCAGACGTGTGCTCTTCCGATCTGTCTCCCAGCTTCTACTTTC	NGS
mHao1_g2_OF7_F	ACACGACGCTCTTCCGATCTGCTCTCTATACTCACCAAGC	NGS
mHao1_g2_OF7_R	CTGGAGTTCAGACGTGTGCTCTTCCGATCTTTACTAACCACTGTGCTGTC	NGS
P5-i5-BC	AATGATACGGCGACCACCGAGATCTACACNNNNNNNNACACTCTTTCCCTACACGACGCTCTTCCGATCT	NGS
rP7-i7-BC	CAAGCAGAAGACGGCATACGAGATNNNNNNNNGTGACTGGAGTTCAGACGTGTGCT	NGS
SaCas9_F	AAGCCATCCCTCTGGAAGAT	qPCR
SaCas9_R	TGCCCTTCTTGCTGTTTTCT	qPCR
mHao1_F	ACCCCTTTTCCTTCATTGCT	qPCR
mHao1_R	TTGCCCAAACACATTTTCAA	qPCR
β-actin_F	ACGGCCAGGTCATCACTATTG	qPCR
β-actin_R	CAAGAAGGAAGGCTGGAAAAGA	qPCR
mAgti3F	GAGACACCGGAATCATCGTT	Genotyping
Neo1600	AGCTCATTCCTCCCACTCAT	Genotyping
mAgte4R	GACCAGGAACAGCAATACCG	Genotyping

Supplementary References

1. Doench, J. G. *et al.* Rational design of highly active sgRNAs for CRISPR-Cas9-mediated gene inactivation. *Nat. Biotechnol.* **32,** 1262–1267 (2014).
2. Hsu, P. D. *et al.* DNA targeting specificity of RNA-guided Cas9 nucleases. *Nat. Biotechnol.* **31,** 827–832 (2013).
